# Successful Administration of Preoperative Botox for Giant Omphalocele Repair With Ultrasound Guidance

**DOI:** 10.7759/cureus.37850

**Published:** 2023-04-19

**Authors:** Arthur J Armijo, Joshua Calvano, Nicolas T Thomason, Christopher Arndt, Anil K Shetty, Dominick Byrd, Ricardo Falcon, Timothy R Petersen, Codruta Soneru

**Affiliations:** 1 Anesthesiology, Rocky Vista University College of Osteopathic Medicine, Parker, USA; 2 Department of Anesthesiology and Critical Care, University of New Mexico School of Medicine, Albuquerque, USA; 3 Department of Surgery, University of New Mexico School of Medicine, Albuquerque, USA

**Keywords:** chatgpt, subfascial tissue expanders, anterior abdominal wall reconstruction, botox, botulinum toxin, omphalocele

## Abstract

We present a case of a four-year-old male with a history of giant omphalocele who underwent ultrasound-guided Botox injection to bilateral anterior abdominal wall musculature in preparation for definitive repair. Botox administration was successfully combined with preoperative subfascial tissue expanders to achieve definitive midline closure of the anterior abdominal wall defect. Our experience suggests that Botox can be safely used as part of the treatment plan for giant omphalocele repair.

## Introduction

An omphalocele is a congenital defect in which abdominal contents protrude outside the abdominal wall into an external sac [1]. This occurs between six to 10 weeks of gestation, with an incidence of 1 in 4,000-6,000 [1]. Giant omphalocele (GO), defined as a defect >5-6cm that involves most or all of the liver, typically requires staged surgical closure and nonoperative delayed closure. However, these methods have frequent hernias (12-56%), morbidity (respiratory and wound complications), and high cost [2].

Recently, Botox (botulinum toxin A [BTA]) has been safely used for abdominal wall reconstruction in pediatric patients [2,3]. Botox administration causes laxity of the abdominal wall musculature and decreases tension on the fascial closure, facilitating midline abdominal wall closure [4]. To our knowledge, this is the first description of a combined preoperative sub-fascial tissue expander and BTA injection for definitive GO repair.

This article was previously presented as a poster at the 2022 Western Anesthesia Residents Conference on April 30, 2022.

## Case presentation

A four-year-old male presented with GO, prematurity, chronic lung disease, tracheostomy, previous vent dependence, pulmonary hypertension (HTN), hypothyroidism, and gastroesophageal reflux disease (GERD). Tissue expanders had been placed four months prior to presentation and there had been two incidents of deflation of the left subfascial tissue expander. In order to optimize conditions for midline closure of the large anterior abdominal wall defect, the decision was made to use BTA in addition to the subfascial tissue expanders.

**Figure 1:**
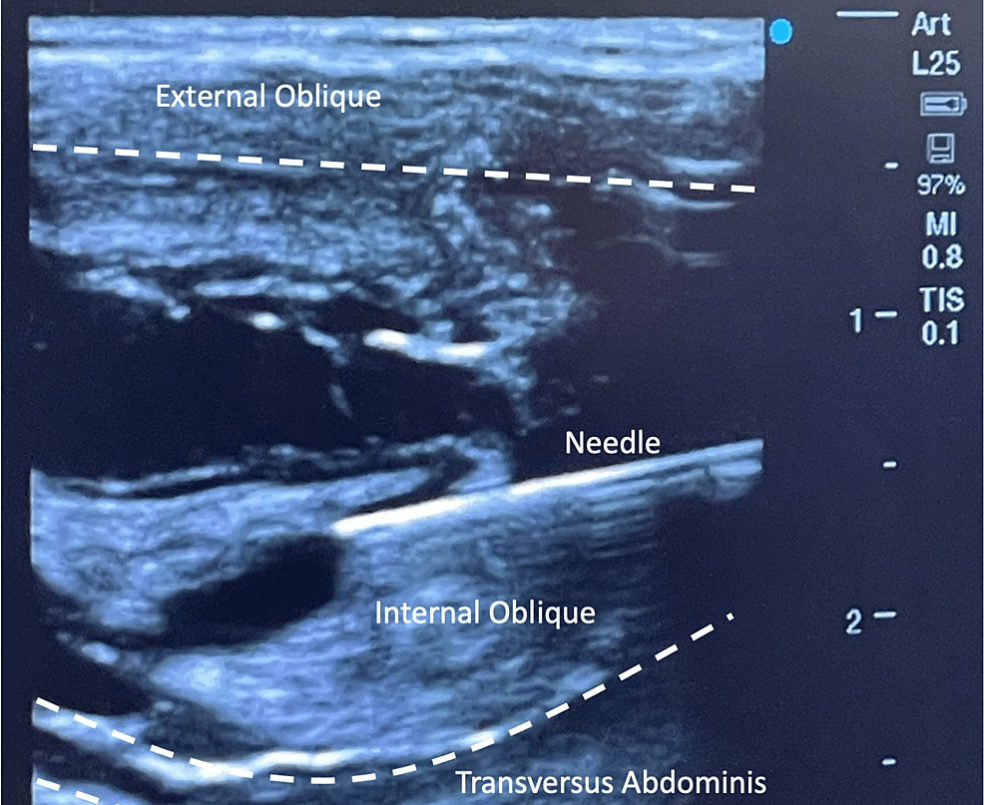
Shown is evidence of needle placement into the anterior abdominal wall musculature for injection of Botulinum Toxin A, utilizing ultrasound guidance.

The patient underwent BTA injections under general anesthesia, with the toxin reconstituted (100 units with 50 mL of saline) and injected into the transversus abdominis, internal oblique, and external oblique muscles using a 22G Pajunk needle and ultrasound guidance (Figure 1). A total of 25 units of BTA were carefully injected intramuscularly into each muscle on the left and right sides. The patient tolerated the procedure well. Following the BTA injections, the patient was positioned supine and the left subfascial tissue expander was inflated with 225 mL of saline using the remote port. The complex anterior abdominal wall reconstruction and closure were performed six weeks later, with a two-week delay due to coronavirus disease 2019 (COVID-19) infection. The patient had a successful postoperative course, requiring ventilator support for less than 48 hours. The patient has remained complication-free for over nine months as of the time of publication.

## Discussion

In surgical treatment of GO, lateral retraction and relative hypertonia of abdominal muscles lead to high rates of closure failure, morbidity, and increased cost [2]. The use of a relaxing agent, such as botulinum toxin, could potentially facilitate expedited closure. Current literature supports the safe and effective use of preoperative BTA injections for the induction of muscle relaxation [2].

Our case demonstrates the successful use of BTA in the definitive treatment of a pediatric patient with GO. The patient had no complications secondary to the BTA injections and had an uneventful surgical recovery. BTA acts by inhibiting the release of acetylcholine, resulting in muscular flaccid paralysis. The toxin takes effect within two weeks, with peak effect in four to six weeks and a gradual decline over three months [4,5]. BTA has also been shown to reduce inflammation and scarring by inhibiting the release of glutamate and substance P [5]. This mechanism may help to reduce postoperative pain. Several studies have reported the safe and effective use of BTA as an adjunct in the repair of complex abdominal wall defects in both adult and pediatric patients [1-5]. BTA injections result in a reversible flaccid paralysis of the lateral abdominal wall musculature allowing for apposition of midline fascia and reducing tension on the fascial repair, decreasing intraabdominal pressure, and mitigating the risk of postoperative complications [4].

Our patient had a mesh and partial closure. Botox and tissue expanders were useful adjuncts for the repair of a GO in a patient with complex medical issues. Previous literature [1-5] reports the use of Botox or tissue expanders for successful abdomen wall reconstruction but no reported use together. 

## Conclusions

Our case highlights the successful combined use of BTA and subfascial tissue expanders in the staged repair of GO. This patient case demonstrates an example in which BTA can be safely administered to the abdominal wall musculature in a pediatric patient with respiratory compromise and ultrasound guidance can be used for effective delivery. This combination provided a tension-free fascial closure of the anterior abdominal wall. Future studies with case controls are necessary to understand the risks and benefits of this combination of therapy. 
